# The role of C-reactive protein in innate and acquired inflammation: new perspectives

**Published:** 2016-09-05

**Authors:** JoAnn Trial, Lawrence A. Potempa, Mark L. Entman

**Affiliations:** 1 Division of Cardiovascular Sciences and the DeBakey Heart Center, Department of Medicine, Baylor College of Medicine, Houston, Texas, 77030, USA; 2 College of Pharmacy, Roosevelt University, Schaumburg, Illinois, 60173, USA

**Keywords:** C-reactive protein, hsCRP assay, macrophage polarization

## Abstract

The participation of C-reactive protein (CRP) in host defense against microorganisms has been well described. More controversial has been its role in chronic conditions such as cardiovascular disease. Our recent publications explain the reasons for some of the confusion concerning CRP as a risk factor for disease and whether it is pro-inflammatory or anti-inflammatory. We found that two isoforms of CRP, pentameric (pCRP) and monomeric (mCRP), on microparticles (MPs), were not measureable by standard clinical assays. When we investigated MPs by imaging cytometry in plasma from controls versus patients with peripheral artery disease, we found that MPs from endothelial cells bearing mCRP were elevated. This elevation did not correlate with the soluble pCRP measured by high-sensitivity CRP assays. The data suggest that detection of mCRP on MPs may be a more specific marker in diagnosis, measurement of progression, and risk sensitivity in chronic disease. In an in vitro model of vascular inflammation, pCRP was anti-inflammatory and mCRP was pro-inflammatory for macrophage and T cell polarization. When we further investigated pCRP under defined conditions, we found that pCRP in the absence of a phosphocholine ligand had no inflammatory consequences. When combined with phosphocholine ligands, pCRP signaled through two Fcγ receptors (FcγRI and FcγRII) via phosphorylation of spleen tyrosine kinase (pSYK) to activate monocytes. Phosphocholine itself, when bound to pCRP, induced a congruent M2 macrophage and Th2 response. Phosphocholine is also the head group on the lipid phosphatidylcholine, which can become oxidized. Liposomes bearing oxidized phosphatidylcholine without pCRP promoted a uniform M1 macrophage and Th1 pro-inflammatory response. When oxidized liposomes were bound to pCRP, there was a disjunction in the macrophage and T cell response: monocytes matured into M2 macrophages, but the T cells polarized into a Th1 phenotype. The CRP-bound liposomes signaled monocytes via FcγRII to promote an anti-inflammatory M2 macrophage state, whereas the lack of FcγR on T cells allowed their liposome-induced polarization to a pro-inflammatory Th1 phenotype unopposed by the contribution of the pCRP/FcγR interaction. Different isoforms of CRP and its binding to complex ligands may determine its biological activities and their contribution to inflammatory states.

## Introduction

CRP is an ancient molecule that existed prior to the evolutionary advent of complement or the acquired immune system ^[1]^. It had a role, now maintained even in higher organisms, of nonself recognition mediating innate immunity ^[2]^. It has been suggested that the functions of CRP became less essential with the development of more complex immunity ^[3]^, and yet there are few species that have lost the ability to make it, and no known human cases of genetic absence. It is part of the innate immune rapid response to infectious organisms, especially in overwhelming infections such as sepsis. In sterile tissue damage, it is also one part of the overall inflammatory response, in which it may have multiple, even opposing, roles depending on its molecular form. In this Research Highlight, we discuss our new findings concerning the different structural forms of CRP, their presence in peripheral artery disease, and their mechanism of action ^[4, 5]^.

**Figure 1:**
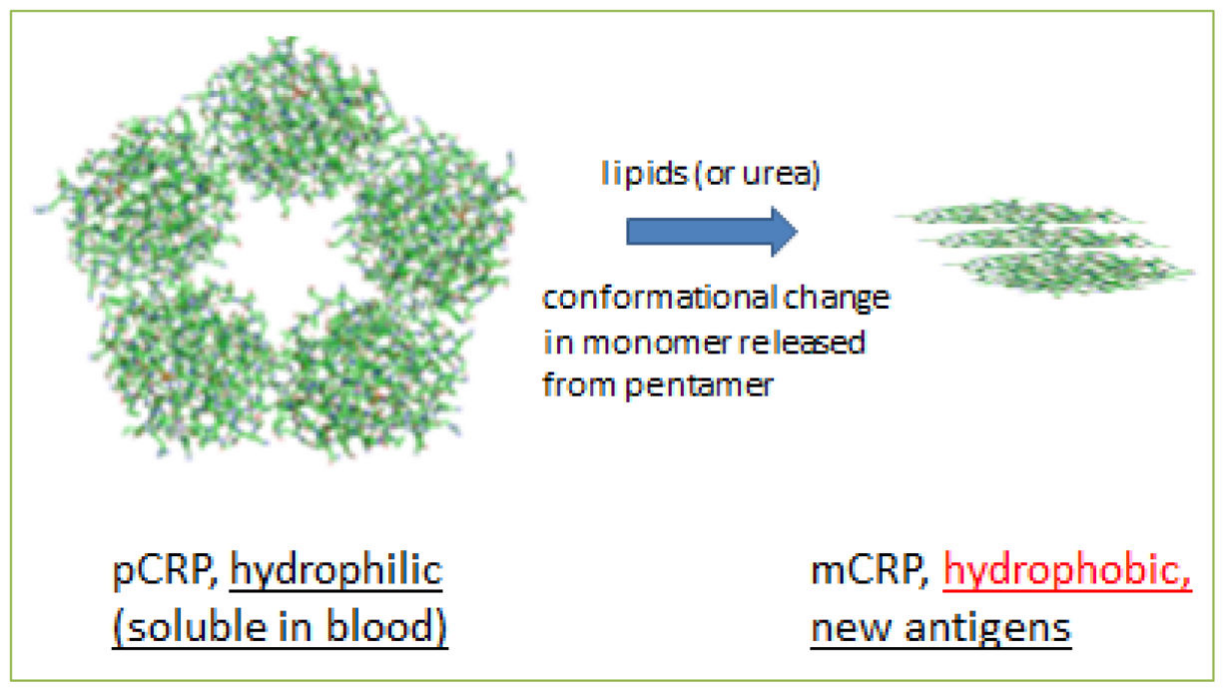
Molecular models of CRP isoforms The pentameric form of CRP (pCRP), which exposes hydrophilic residues, is induced by lipids or urea to relax into hydrophobic monomeric subunits (mCRP) that expose novel antigenic residues. These altered monomers may associate into dimers or trimers, as illustrated.

CRP found in the circulation is produced and secreted by the liver as a soluble pentamer (pCRP) of five identical monomeric subunits ^[6]^. When pCRP is exposed to conditions that separate individual subunits, the resultant monomeric CRP (i.e. mCRP) undergoes a conformational rearrangement that makes it substantially less soluble and new antigens are exposed ^[7]^ (Figure 1). However, the exact structure of mCRP is unknown: it may form dimers or trimers with the altered conformation. In looking for naturally occurring substances that could affect the dissolution of pCRP into mCRP, we and others have found that this process can be aided by lipids ^[4, 8]^. In the body, lipids derived from cell membranes, apoptotic bodies, microparticles, or LDL cholesterol particles can subserve that function ^[9–11]^. To study individual CRP monomers *in vitro*, the pentamer can be relaxed by denaturants such as urea, detergents, and lipid monolayers or bilayers; the resultant monomers cannot reassemble, and stay in the lipid environment because of their hydrophobicity. Individual CRP subunits dissociated from those found in the pentameric configuration are structurally and functionally distinct and are described as “modified, monomeric” CRP or “mCRP.” The released monomeric subunits reveal new epitopes for antibody recognition and monoclonal antibodies that clearly distinguish pCRP or mCRP ^[12]^ were used in the studies we describe herein. As described in this report, mCRP antigen in plasma can be detected only as a complex with lipid containing microparticles. mCRP, both as an isolated protein and as a lipid complex, is now widely recognized as a biologically active form of “CRP” with potent pro-inflammatory activities in contradistinction to pCRP ^[13]^.

To identify naturally occurring substances that could elicit the release of mCRP from pCRP, we evaluated the role of microparticles derived from TNF-α stimulated cultured human cardiac endothelial cells or measured in plasma from patients with peripheral artery disease. Both pCRP and mCRP have been found on the surface of microparticles, which are ~100–1000 nm vesicles that have budded from the cell surface and contain part of the outer cell membrane as well as internal cellular components. Thus, just as artificial lipid membranes have been shown to promote the relaxation of the CRP pentamer into its component monomers, a similar dissociation of pCRP into mCRP has been reported to occur on naturally produced microparticles ^[11]^. The particular relevance to these findings in all clinical and diagnostic studies of CRP-involved inflammatory disorders is that, while pCRP is freely soluble and easily quantifiable in plasma, the mCRP isoform is not freely soluble and is only found associated with particles or in atheromas. Identifying and quantifying mCRP in plasma thus requires specialized reagents and techniques that address the confounding contributions of lipids to measurement techniques. This communication summarizes these findings and discusses their potential biological significance to immunobiology and, potentially, to pathophysiology.

## Potential clinical relevance

**Figure 2:**
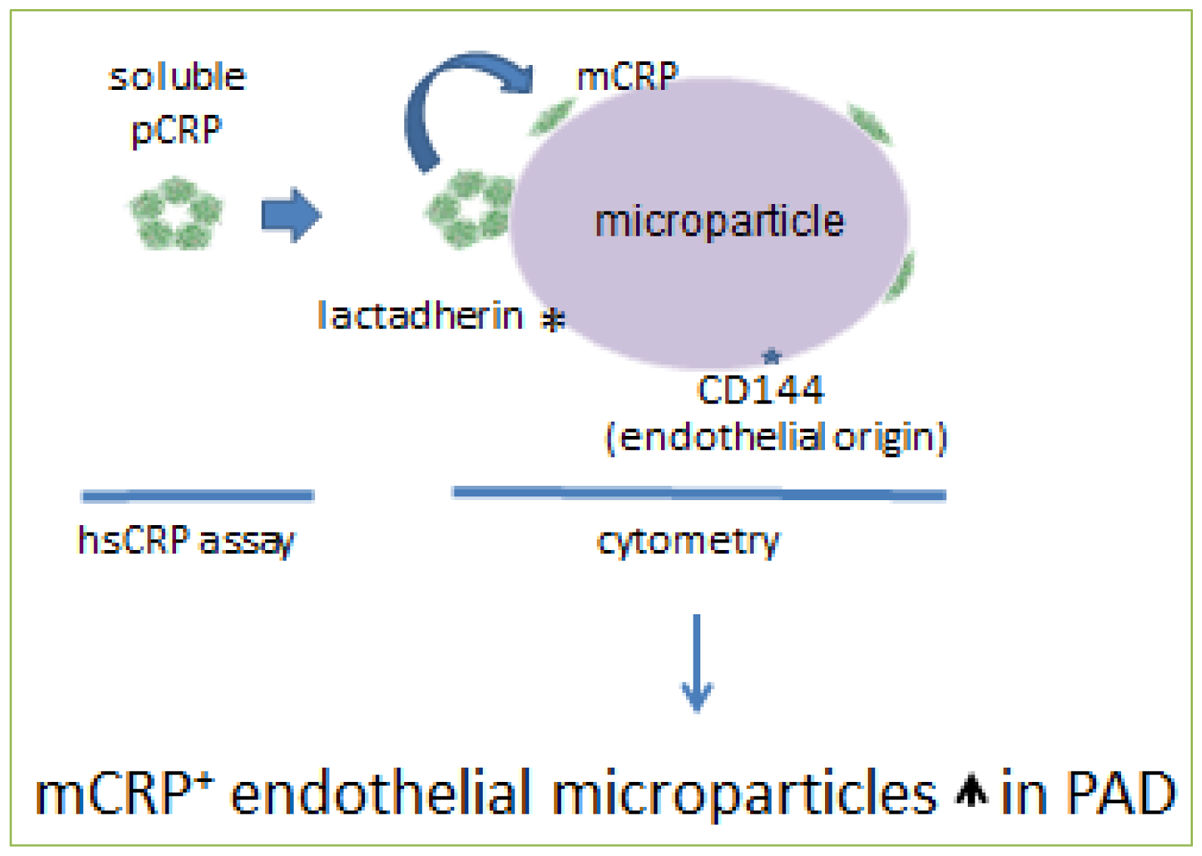
Schema of results from different types of analysis for CRP isoforms Soluble pCRP in blood is the only form of CRP measurable by an hsCRP assay. pCRP and mCRP on microparticles were found by imaging flow cytometry, which identified microparticles by lactadherin binding and endothelial cell of origin by anti-CD144 binding. The number of endothelial microparticles bearing mCRP was elevated in PAD patients.

We initially asked if standard clinical high sensitivity CRP (hsCRP) assays could detect mCRP or pCRP on microparticles, and found that they could not, even when the microparticles were deliberately loaded with either form of CRP. They also could not detect urea-solubilized mCRP. hsCRP assays, therefore, may only measure the portion of total CRP in plasma that is in the soluble pentameric form (soluble pCRP). However, using imaging flow cytometry and antibodies that distinguish between pCRP and mCRP, we were able to measure both forms of CRP on microparticles, revealing a compartment of CRP not currently measured in clinical blood samples (Figure 2). We then investigated the presence of both CRP isoforms on microparticles in the plasma of control donors versus that of patients with peripheral artery disease (PAD). For that study, we also used antibodies defining microparticles originating from different cell types. We hypothesized that cell specificity might detect the site of specific pathophysiologic events. Consistent with PAD being a vascular disease, we found a large increase in microparticles from endothelial cells in patients compared to controls. Further, there were more endothelial microparticles carrying mCRP, but no increase in those bearing pCRP, in patients (Figure 2). By contrast, there was no increase in microparticles of platelet or monocyte origin. We postulated that the increase in microparticles carrying mCRP could have resulted from an enhanced conversion of pCRP to mCRP on the endothelial microparticles of patients.

**Figure 3:**
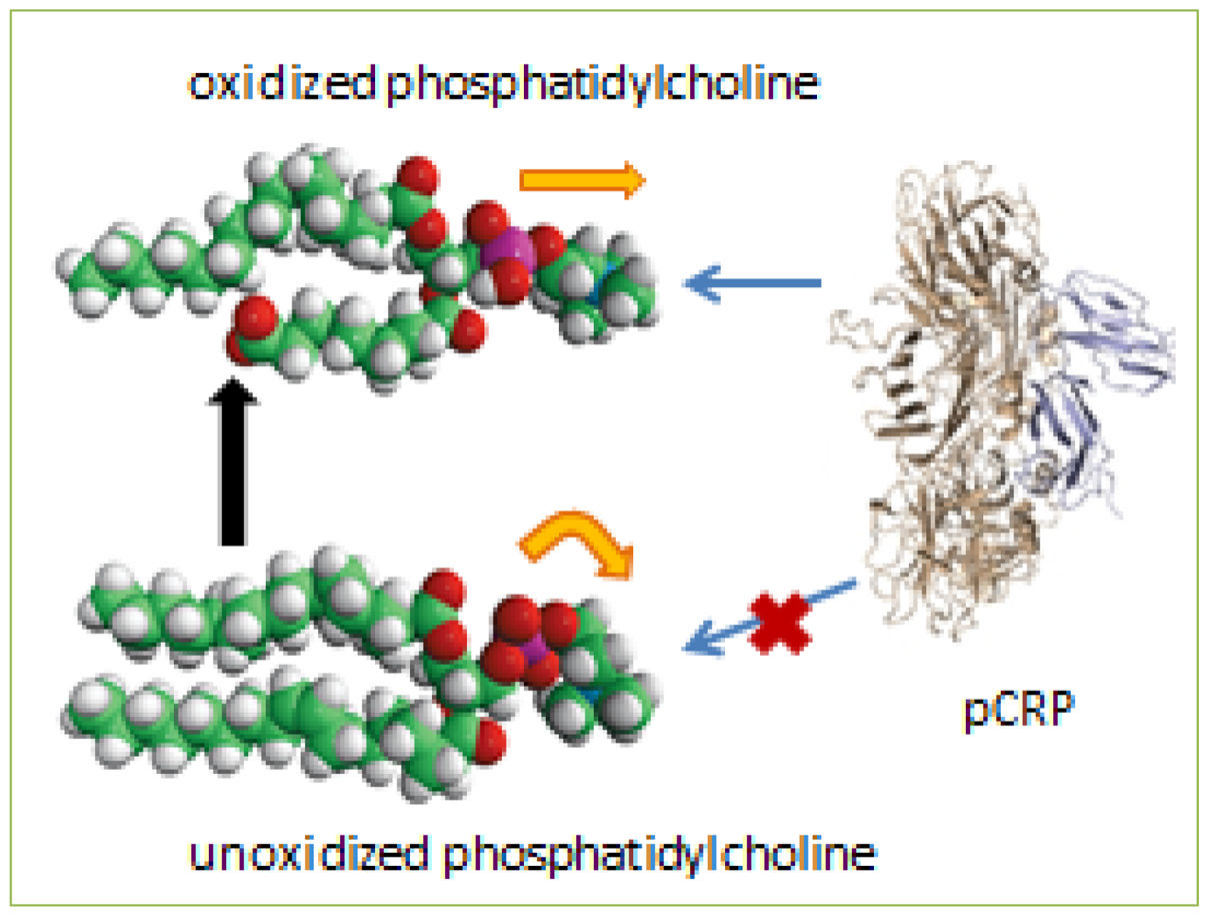
Molecular model of oxidation of phosphatidylcholine The model shows the truncation of one fatty acid chain by oxidation (black arrow), as well as the straightening of the phosphocholine head group (orange arrows) allowing CRP to bind. The CRP illustrated is the pentamer shown perpendicular to its planar orientation (on edge).

This possibility was further suggested by our finding that the number of mCRP-bearing endothelial microparticles was also highly correlated with the amount of low-density lipoprotein cholesterol (LDL) found in the patient blood. Although we did not measure the oxidation state of the LDL in patient plasma, CRP can only bind to oxidized (as opposed to unoxidized) phospholipids ^[14]^(Figure 3), and lipids are a critical part of the mechanism for dissociating pCRP into mCRP ^[8, 15]^. Thus, higher levels of LDL, and possibly its oxidation, may be a component of processes that include greater conversion of pCRP to mCRP on microparticles.

Oxidized LDL is a known risk factor for cardiovascular disease ^[16, 17]^, even in healthy individuals ^[18]^, and so further investigations into these relationships is warranted. It is vital to find the best indicator of disease progression for clinical use, but it is also important to reveal the biological bases so that rational interventions can be designed. These data suggest that microparticle-bound mCRP is an important biological marker and that one important ligand for mCRP could be oxidized lipoprotein bound to LDL on endothelial cells. The interaction of CRP with LDL and other factors in atherosclerosis has recently been reviewed, although without consideration of CRP isoforms ^[19]^.

## Potential biological relevance

**Figure 4:**
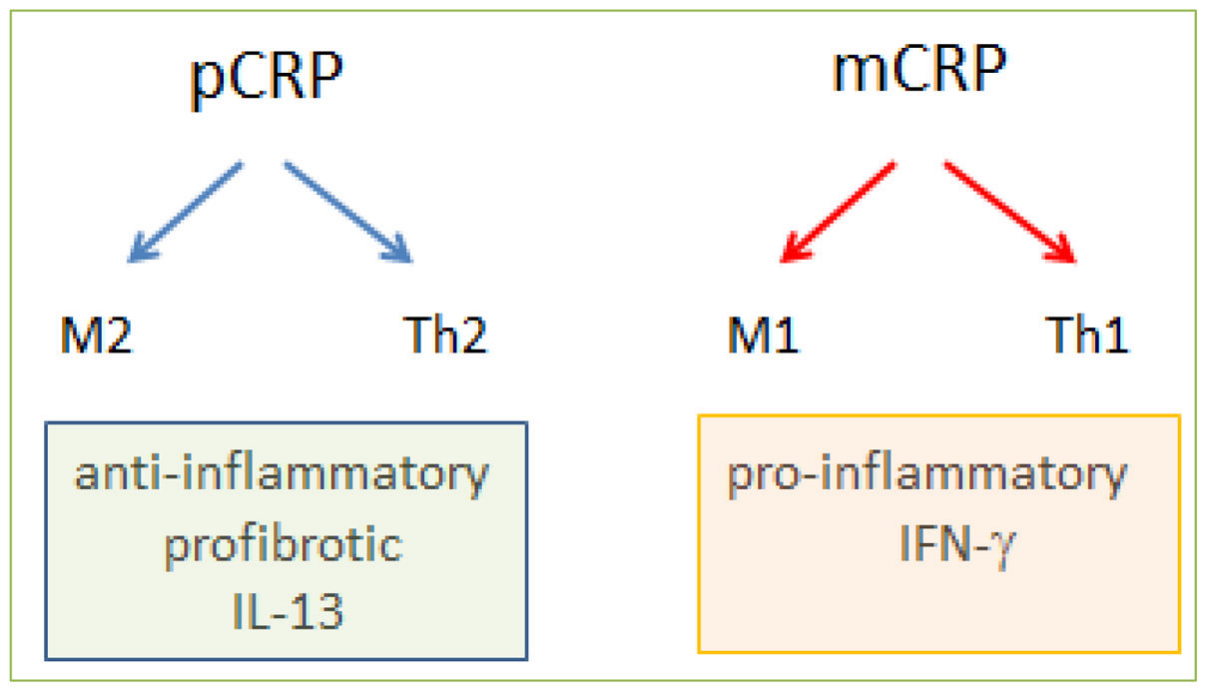
Polarization of monocytes and T cells by CRP isoforms In the TEM assay, performed in the presence of fetal bovine serum, the addition of pCRP causes the maturation of monocytes into M2 macrophages and T cells polarize to a Th2 phenotype. This results in an anti-inflammatory and profibrotic environment characterized by the production of interleukin-13 (IL-13). The addition of urea-solubilized mCRP results in M1 macrophage maturation and Th1 polarization, resulting in a pro-inflammatory environment characterized by the production of interferon-γ (IFN-γ).

To investigate further the biological relevance and consequences of the two forms of CRP, we employed an in vitro model of leukocyte transmigration across human cardiac microvascular endothelium (transendothelial migration, TEM). We observed the consequences of pCRP versus mCRP on the maturation of monocytes into polarized macrophages. We assessed M1 (proinflammatory) and M2 (anti-inflammatory, profibrotic) macrophage polarization and T cell polarization after TEM in the presence of the two CRP isoforms. Previous reports of CRP’s biological activity had concluded that it was proinflammatory. Our investigation found that pCRP was anti-inflammatory (and profibrotic), whereas mCRP was the pro-inflammatory isoform (Figure 4).

T cells can drive macrophage polarization by means of cytokines. For example, Th1 cell production of IFN-γ polarizes macrophages to an M1 phenotype, whereas Th2 cell-derived IL-13 promotes the corresponding M2 macrophage polarization. Conversely, macrophage polarization in an early innate immune response can influence T cell polarization, and so the two types of immunity are generally aligned. Therefore, as expected, the M2 macrophage profibrotic response to pCRP was accompanied by a Th2 T cell response (also anti-inflammatory and profibrotic). Likewise, mCRP induced not only an M1 macrophage response, but a matching Th1 response (pro-inflammatory) (Figure 4). Thus, elements of the innate immune response (monocyte/macrophage) responded in the same direction of polarization as members of the acquired immune system (T cells) under these conditions. Other reports have also indicated that purified CRP (likely in the pentameric form) is not pro-inflammatory ^[20–22]^, whereas mCRP is ^[23–25]^. Further research to determine the dominant response when both isoforms are present on the same microparticle is clearly necessary to understand more fully the consequences for disease states.

## CRP binding to ligands is essential for bioactivity

We have recently extended our studies of the pentameric form of CRP into questions concerning its ligands and mode of signaling ^[5]^. To make our model of endothelial transmigration more defined, we used a serum-free environment with two different CRP ligands that are relevant to cardiovascular disease: phosphocholine and an oxidized phospholipid.

Phosphocholine is a terminal head group of the lipoteichoic acid component of the cell walls of some Gram positive bacteria, including *Streptococcus pneumoniae*. This and other organisms are known to infect the heart ^[26]^ or have been found in atheromatous plaques ^[27]^. Phosphocholine is also the head group on the lipid phosphatidylcholine. The exposure of the PC group on such lipids can be regulated by oxidation, which straightens the PC conformation and makes it available for CRP binding (Figure 3). We modeled the interaction of CRP with oxidized phosphatidylcholine by making multilamellar liposomes with unoxidized phosphatidylcholine, to which CRP does not bind, mixed with a small percentage of a partially oxidized phosphatidylcholine, as might be foundin LDL.

**Figure 5:**
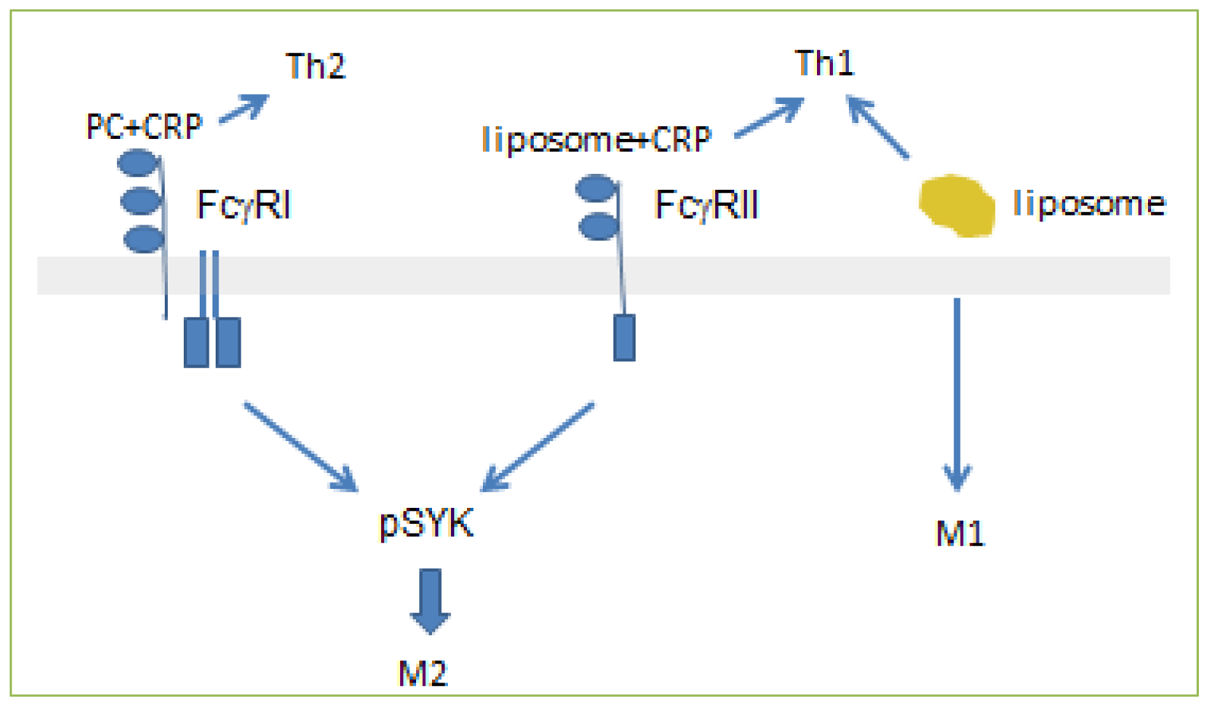
Polarization of monocytes and T cells by CRP bound to different ligands and liposomes alone PC alone does not signal immunocytes to polarize, and so is not represented in this schema. PC plus CRP signals via FcγRI to phosphorylate spleen tyrosine kinase (SYK, pSYK) and promote M2 macrophage maturation as well as a Th2 response. Liposomes plus CRP also activate SYK, but via FcγRII. However, this signal pathway for macrophages does not affect the T cell response, which is toward a Th1 phenotype. Liposomes without CRP induce a uniform proinflammatory response in both macrophages (M1) and T cells (Th1). Reprinted with permission [5].

We found that when we used serum-free conditions in our assay, CRP without ligands had no effect on macrophage polarization. However, when bound to phosphocholine, CRP promoted the M2 polarization of macrophages and Th2 polarization of T cells, as would be expected for congruent innate and acquired immune responses. By contrast, when CRP was bound to liposomes containing oxidized phosphatidylcholine, a different pattern emerged. Although M2 differentiation of macrophages was induced, the T cells in the assay became polarized to a Th1 phenotype, with production of proinflammatory IFN-γ; i.e., there was a “mismatch” between the macrophage and T cell responses. Oxidized lipids alone have been reported to induce M1 macrophage and Th1 T cell responses ^[28, 29]^, which could explain the Th1 polarization in our model. We discovered that the disjunction between the macrophage and T cell responses in our liposome studies was caused by rapid signaling of the CRP-liposome combination through Fcγ receptors on the monocytes. T cells do not express Fcγ receptors, and therefore retained their Th1 response. Both FcγRI and FcγRII signaled through spleen tyrosine kinase (SYK), a transcription factor responsible for cellular activation. The subsequent M2 macrophage response, which resulted in the production of autocrine IL-13, was insufficient to oppose the oxidized lipid-induced polarization of T cells into a proinflammatory Th1 phenotype (Figure 5). In this case, the macrophage polarization to the M2 phenotype preceded and was independent of the T cell response and could not subsequently influence it. Thus, the evolved response to infectious microorganisms, represented by CRP binding to phosphocholine moieties expressed on the surface of the microorganisms, may lead to resolution of inflammation, represented by an M2/Th2 response, whereas the proinflammatory influence of oxidized lipids may be ameliorated by pCRP in the case of macrophages, but not T cells. This may lead to the coexistence of pro- and anti-inflammatory/profibrotic conditions in the atherosclerotic plaque that has been described by others ^[30, 31]^.

**Figure 6:**
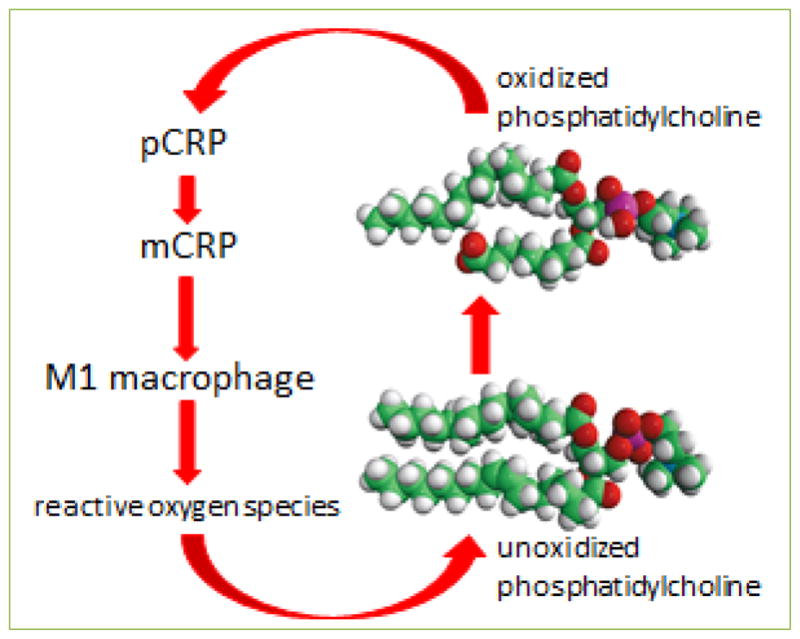
Hypothetical feed-forward loop of mCRP action on macrophages and lipids The differentiation of monocytes into M1 macrophages by mCRP may result in the production of reactive oxygen species that oxidize lipids such as phosphatidylcholine. The oxidized phosphatidylcholine, for example on low density lipoprotein cholesterol particles, may generate more mCRP from pCRP on the particles.

The binding of pCRP to oxidized lipids relaxes the pentamer into mCRP subunits, which promotes the maturation of monocytes into M1 proinflammatory macrophages. M1 macrophages generate reactive oxygen species that can oxidize lipids. We speculate that this sequence of events may represent a feed-forward effect that promotes further generation of mCRP from pCRP on lipid membranes (Figure 6) and maintains the pro-inflammatory state.

## Evolutionary advantages of multiple CRP isoforms

pCRP binds phosphocholine head groups on bacterial cell wall components and phospholipids in blood and on cells. Even before the evolution of the acquired immune system and complement, it likely served as a way to remove microorganisms and dead cells from the body. It was then integrated into more complex immune systems as they evolved, and now may serve a variety of functions. The pentameric form may contribute first as an opsonin, to remove nonself or damaging self components, and then as a regulator of the stimulated inflammatory response, dampening inflammation by inducing an M2/Th2 response. If the threat to survival remains paramount, however, the oxidized phospholipids created and expressed during the acute phases of the host defense response may contribute to the disruption of the pCRP conformation to produce mCRP, which is predominantly expressed as a complex with lipids.

In a tightly controlled, productive inflammatory response, generation of mCRP could contribute important protection against the substance that threatened health and homeostasis. It may normally amplify the initial response to a threat by inducing inflammation transiently, and then be cleared along with the instigating substance. If, however, the instigator remains present chronically, then the continuous generation of mCRP may run counter to the re-establishment of homeostasis and lead to a persistent inflammatory disease.

## Conclusions

The implications of these findings for human biology are two-fold. First, the clinical measurement of CRP by hsCRP assays does not reveal the entire picture of the role that CRP plays in chronic illness. Whether or not the inclusion of further aspects of CRP biology such as microparticle measurements (as we have reported) will aid in diagnosis or treatment of disease must rely on many further studies. Second, the understanding of the role of CRP isoforms and their interactions with oxidized lipids in atherosclerosis and other cardiovascular diseases should be expanded.

CRP was originally isolated as a lipid-associated protein ^[32]^. When lipid was removed for better protein analyses, CRP biochemical characteristics such as solubility, electrophoretic mobility, apparent isoelectric point and size, were changed ^[33]^. As the field of CRP research has evolved over the past 50 years, the delipidated protein became the predominant focus of structural, functional, and clinical studies that currently dominate the understanding of this protein as a biologically relevant substance. Our current work gives us a new appreciation of the different forms of CRP, including the lipid-associated entities, and represents a key turning point in elucidating the multiple biological roles of CRP in health and disease.

## Abbreviations

CRP: C-reactive protein
FcγR: receptors for the Fc portions of IgG
hsCRP: high sensitivity CRP assay
IFN-γ: interferon-γ
IL-13: interleukin-13
M1: macrophage type 1
M2: macrophage type 2
PC: phosphocholine (phosphorylcholine)
TEM: transendothelial migration
Th1: T helper type 1
Th2: T helper type 2
SYK: spleen tyrosine kinase
